# Genotyping of *Candida albicans* isolated from animals using 25S ribosomal DNA and ALT repeats polymorphism in repetitive sequence 

**DOI:** 10.18502/cmm.4.4.381

**Published:** 2018-12

**Authors:** Armina Dalvand, Farzad Katiraee, Raziallah Jafari Joozani, Hojjatolah Shokri

**Affiliations:** 1 Department of Pathobiology, Faculty of Veterinary Medicine, University of Tabriz, Tabriz, Iran; 2 Department of Clinical Sciences, Faculty of Veterinary Medicine, University of Tabriz, Tabriz, Iran; 3 Department of Pathobiology, Faculty of Veterinary Medicine, Amol University of Special Modern Technologies, Amol, Iran

**Keywords:** Animal, Antifungals, Candida albicans, Genotyping, Repetitive sequences

## Abstract

**Background and Purpose:**

*Candida albicans* is the most prevalent *Candida* species isolated from animals. Candidiasis can be systemic in animals or may affect a single organ, such as the mouth, urinary tract, and skin. The aim of the present study was to determine the genetic diversity of *C. albicans* isolated from different animals and investigate the presence of a relationship between host specificity and genetic typing of *C. albicans*.

**Materials and Methods:**

For the purpose of the study, DNA extraction was performed on 27 clinical isolates of *C. albicans* obtained from animals. Subsequently, they were subjected to 25S ribosomal DNA amplification and ALT repeats in repetitive sequences (RPSs). The minimum inhibitory concentrations of fluconazole, ketoconazole, clotrimazole, nystatin, amphotericin B, and caspofungin were determined using the microdilution method based on the Clinical and Laboratory Standards Institute M27-S4 standard.

**Results:**

Out of 27 *C. albicans* strains, 11, 6, 5, and 5 cases were recognized as genotypes A (40.8%), E (22.2%), B (18.5%), and C (18.5%), respectively, through amplification using AS-I, which revealed 17 different types of *C. albicans*. By combining the two typing methods, 27 *C. albicans* strains were finally divided into 22 genotypes.

**Conclusion:**

Different genotypes showed genetic diversity among the *C. albicans* strains isolated from animal sources. The results revealed no special genotype relationship according to the host, anatomical source of isolation, and antifungal susceptibility.

## Introduction

*Candida* infections are the most important and prevalent fungal infections in humans. *Candida* species are present in humans and animals as commensal microbial flora or opportunistic microorganisms. These species can be found in the skin and gastrointestinal tract of healthy individuals [1, 2]. *Candida albicans* is medically important since it accounts for opportunistic infections in HIV-infected and burn patients, as well as those undergoing organ transplantation, chemotherapy, and radiotherapy [3-5]. 

Furthermore, *C. albicans* can cause a wide spectrum of clinical candidiasis, including localized or systemic fungal infections, such as vulvovaginal or oral infections [6]. Accordingly, it is the most prevalent *Candida* species isolated from such animals as cats, dogs, fowls, swine, cattle, and horses. In animals, candidiasis can be systemic or may affect a single organ, such as urogenital tract infections or cutaneous candidiasis. If the host immune system is severely compromised; for example, in premature newborn animals, *C. albicans* can cause severe infections with a high mortality rate [7-9]. 

Similar to humans, the most susceptible animals to candidiasis are those with severe diseases (e.g., cancer and diabetes), newborns, and animals with long-term antibiotic therapy [10-12]. The prevalence of *Candida* infections is on a rapidly growing trend. *Candida* species are frequently isolated from the clinical samples of different animals using several molecular biology-based techniques. These techniques include polymerase chain reaction (PCR), Southern blotting, random amplification of polymorphic DNA (RAPD), and fluorescence in situ hybridization that are adopted for the detection and identification of fungal species in clinical specimens [13]. 

However, to the best of our knowledge, no comprehensive studies have been conducted on *Candida* species isolated from animals in Iran. Molecular diagnostic methods are available; however, there is limited information regarding the genetic diversity of *C. albicans* isolates from animal subjects. Nonetheless, many reports have targeted candidiasis in animals [8, 11, 13, 14]. Little is known about the identity and origin of these fungal strains and the genetic relationships among the isolates obtained from animals. *Candida albicans* is an opportunistic organism, which is present as natural flora in the mucosa. However, it can be transmitted from one person to another, and its infection can be easily transmitted in flocks [15].

Based on biotyping and other molecular typing methods, no significant differences have been reported between human and animal strains in the literature [16, 17]. It is believed that in most of the cases, the infection is caused by the strains present in the body of patients themselves. However, it seems that commensal strains would be replaced by other more pathogenic strains, which may also show resistance to antifungal drugs. 

All domestic animals, including livestock, horses, pigs, dogs, cats, and birds, are sensitive to *Candidiasis *[16]. It is quite probable that these animals could be a carrier of the diseases or a potential source of infection transfer to humans or other animals. In other words, patients with immunodeficiency disorders are prone to a high risk of infection [18]. Therefore, attention should be directed toward the crossover transfer of yeast infections between humans and animals as well as from animals to animals. It is also essential to investigate and compare different human and animal *Candida* strains in several geographical regions.

There are different molecular methods for the clinical diagnosis of Candidiasis. *Candida* species can be distinguished carefully at species level. Determination of 25S ribosomal DNA (rDNA) is one of these methods, which have been used for many years. In this approach, *Candida* species are divided to four genotypes of A, B, C, and E, and genotype D is *C. dubliniensis*. The use of typing methods, which can carefully classify different strains, has been fascinating for researchers since *Candida* species have always shown several genotypes. Chromosomes of *C. albicans* include repetitive sequences called RPS, entailing 172 consecutive repetitive nucleotides referred to as alternative lengthening of telomerase (ALT).

The number of ALTs leads to diversity in the molecular size of RPSs. Accordingly, the molecular identification of different sizes and copy numbers of ALT sequences has become an appealing method for researchers. Previous studies have shown that other species of *Candida* include RPS regions; however, *C. albicans* is more variable and diverse, compared to others [19]. This study involved the genotyping of *C. albicans* strains isolated from animals based on the specification of 25S rDNA types and frequency of ALT in RPS regions using a fast and inexpensive method. 

With this background in mind, the aim of the present study was to determine the genotypes and genetic diversity of *C. albicans* isolated from different animals. This study was also targeted toward the investigation of the presence of correlation between host specificity and *C. albicans* genotypes.

## Materials and Methods

***Strains of Candida albicans***

A total of 27 *C. albicans* strains isolated from clinical animal specimens were used as templates for PCR amplification, 13, 7, and 7 cases of which were isolated from the oral cavity, scale, and vagina, respectively. Out of the 27 samples, 12, 5, 4, 3, and 3 *C. albicans* strains were isolated from dogs, cats, horses, cows, and birds, respectively. 

All strains were maintained at ambient temperature in microtubes containing distilled water at the Mycology Laboratory of the Faculty of Veterinary Medicine, University of Tabriz, Tabriz, Iran. The yeast cells were spread onto yeast peptone extract agar containing 2% glucose, and cultured for 48 h at 30ºC before DNA extraction. The confirmation of *C. albicans* was accomplished by means of PCR-restriction fragment length polymorphism technique using *MspI* restriction enzyme as previously described [20].

***Polymerase chain reaction primers***

The genotypes of *C. albicans* was determined on the basis of 25S rDNA type using the forward primer CA-INT-L (ATAAGG GAA GTC GGC AAA ATA GAT CCG TAA) and reverse primer CA-INT-R (CCT TGG CTG TGG TTT CGC TAG ATA GTA GAT; SBS Genetech Co. Ltd). *Candida albicans* were grouped into five genotypes according to the size of PCR products (Table 1) [21, 22]. Genotype D corresponded to *C. dubliniensis*. In addition, the primer set involving ASaF13 (**ATGTCCGTTGAAGACTGCGCGATGAAAAAT)** and AScR13 (**GATGCAGTTAAATCTCGTTTTTAACAGTG)** were used, which were referred to as AS-I, reflecting the number of ALT in RPS unit [22, 23]. 

***Polymerase chain reaction amplification***

DNA extraction was performed using a physicochemical method previously described [24]. For genotyping on the basis of 25S rDNA and RPS sequences, genomic DNA was amplified in a reaction mixture (25 µl) containing 1.75 mM magnesium chloride, 0.2 mM dNTPmix, 1U Taq DNA polymerase (Fermentase), 50 pmol of primers (i.e., CA-INT-L and CA-INT-R or AS-I), and 2 µl DNA template. 

The PCR conditions included 95°C incubation for 7 min prior to 35 cycles of 94°C for 30 sec, 59°C for 25 sec, 72°C for 30 sec, and 72°C for 5 min. All reactions were amplified using a thermal cycler (Primus MWG Biotech, USA). The PCR products were electrophoresed on 1.5% agarose gel in TBE buffer to identify the genotypes of the yeast isolates. After staining with FLUORODYE (SMOBIO), DNA bands were visualized using an ultraviolet transilluminator, and then photographed.

**Table 1:** Final designated genotypes of *Candida albicans* strains based on ABC and repetitive sequences typing

**No**	**Isolate number**	**Host**	**Age**	**Source**	**ABC types**	**ALT sub groups**	**Final Genotype** *
1	CA2	Dog	J	Oral	A	AS1	A-AS1
2	CA6	Dog	A	Oral		AS1	A-AS1
3	CA8	Bird	N	Oral		AS2	A-AS2
4	CA27	Cattle	A	Skin		AS2	A-AS2
5	CA12	Horse	A	Vagina		AS3	A-AS3
6	CA24	Cat	A	Oral		AS4	A-AS4
7	CA26	Cat	A	Skin		AS5	A-AS5
8	CA25	Cat	A	Oral		AS7	A-AS7
9	CA17	Dog	A	Vagina		AS10	A-AS10
10	CA19	Dog	A	Skin		AS12	A-AS12
11	CA18	Dog	A	Vagina		AS16	A-AS16
12	CA1	Dog	J	Oral	B	AS1	B-AS1
13	CA10	Horse	A	Vagina		AS1	B-AS1
14	CA22	Cat	A	Skin		AS4	B-AS4
15	CA14	Cattle	A	Vagina		AS6	B-AS6
16	CA 23	Cat	A	Oral		AS14	B-AS14
17	CA 4	Dog	A	Skin	C	AS2	C-AS2
18	CA 13	Horse	A	Vagina		AS2	C-AS2
19	CA 16	Bird	N	Oral		AS9	C-AS9
20	CA 11	Horse	A	Vagina		AS13	C-AS13
21	CA 21	Dog	J	Oral		AS14	C-AS14
22	CA 15	Cattle	A	Skin	E	AS8	E-AS8
23	CA 3	Dog	A	Oral		AS9	E-AS9
24	CA 20	Dog	J	Skin		AS11	E-AS11
25	CA 5	Dog	A	Oral		AS15	E-AS15
26	CA 7	Dog	A	Oral		AS15	E-AS15
27	CA 9	Bird	N	Oral		AS17	E-AS17

A: adult, J: juvenile, N: nestling

* (Out of 27 strains, 22 genotypes were found.)

***Antifungal susceptibility***

The minimum inhibitory concentrations (MICs) of fluconazole, ketoconazole, clotrimazole, nystatin, amphotericin B, and caspofungin were determined using microdilution method based on the Clinical *and* Laboratory Standards Institute (CLSI) M27-S4 standard [25, 26]. All antifungal agents were purchased from Sigma Aldrich, Germany. The final concentrations of amphotericin B, nystatin, and caspofungin ranged from 0.03 to 8 µg/ml. Moreover, the final concentrations of fluconazole had a range of 0.5-128 µg/ml, and those of clotrimazole and ketoconazole ranged 0.03-16 µg/ml. 

The broth microdilution plates were sealed and incubated at 35°C for 48 h. Finally, visible MIC endpoints were determined using a mirror. The MIC90 was defined as the minimum concentration required to inhibit the growth of 90% of isolates. The CLSI has recommended an interpretive susceptibility criterion, according to which fluconazole resistance was defined as MIC ≥ 8 µg/ml, while ketoconazole and clotrimazole resistance was considered as MIC ≥ 1 µg/ml. 

Moreover, a MIC breakpoint of ≤ 2.0 µg/ml indicated susceptibility to amphotericin B and nystatin, while a MIC of > 2.0 µg/ml suggested the resistance of isolates. With regard to caspofungin, susceptibility and non-susceptibility were defined as MIC breakpoints of ≤ 1 and > 1 μg/ml, respectively, as previously described and approved by CLSI [27, 28]. The data were analyzed in SPSS software (version 11.5) using descriptive tests and cross tabulation. 

## Results

**Figure 1:**
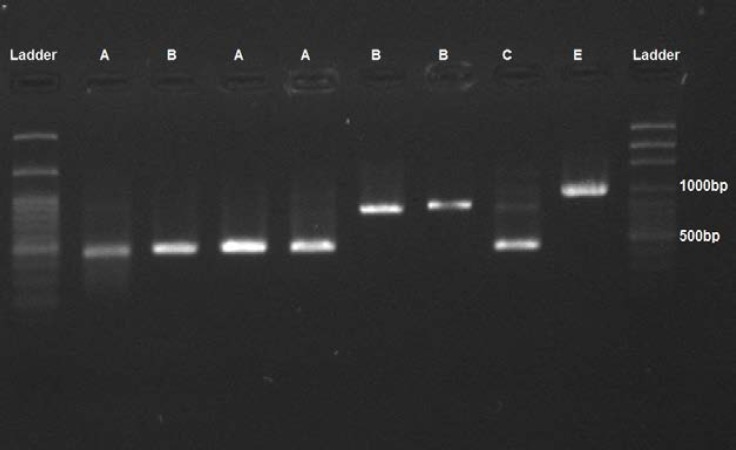
Amplification profiles of polymerase chain reaction (PCR) products of *Candida albicans* isolates in the PCRs targeting 25S rDNA (ABC typing) (Genomic DNA purified from various yeast species was amplified by PCR using CA-INT primer.)

Genomic DNA of the clinical isolates was amplified using CA-INT, and the genotypes were determined on the basis of 25S rDNA (Figure 1). Out of 27 *C. albicans* strains, 11, 6, 5, and 5 cases were recognized as genotypes A (40.8%), E (22.2%), B (18.5%), and C (18.5%), respectively (Table 1); however, no genotype D was found in this study. Accordingly, genotype A *C. albicans* constituted the majority of the isolates, followed by genotype E *C. albicans*. 

**Figure 2:**
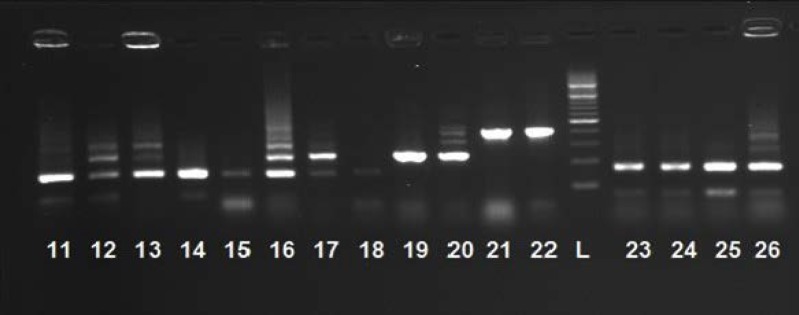
Subtype analysis of *Candida albicans *by repetitive sequences (RPS) primers (Amplification profiles of polymerase chain reaction (PCR) products of *C. albicans* isolates in the PCRs targeting RPSs. Genomic DNA purified from various yeast species was amplified by PCR using AS-I primer.)

The ABC genotypes that were not discriminated by 25S rDNA-based genotyping were analyzed by PCR using AS-I and the electrophoretic patterns of PCR fragments. Some clinical samples with the same 25S rDNA genotype or ABC type (e.g., isolates number 2, 8, and 12) were distinguished by AS-I (Figure 2). 

**Figure 3:**
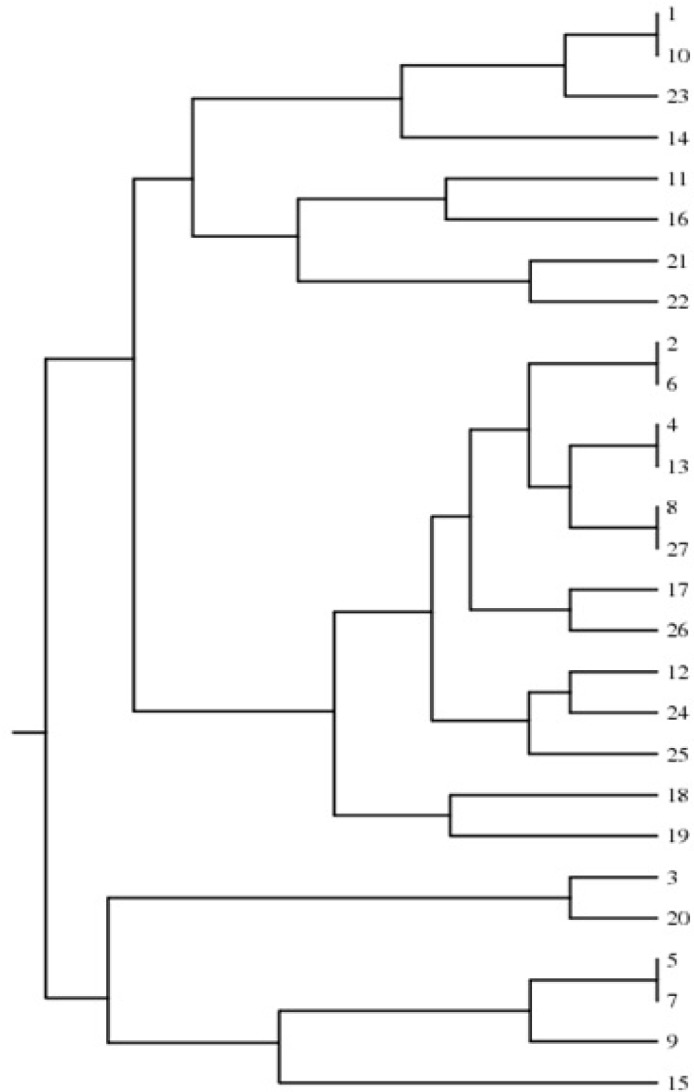
UPGMA dendrogram using the Dice similarity coefficient, genetic variation among *Candida albicans* strains based on 25S rDNA and ALT-based genotyping (22 genotypes were identified out of 27 *C. albicans* isolated from animals.)

The PCR using AS-I revealed 17 different types of *C. albicans *designated as AS-1 to AS-17. Genotypes AS1 and AS2* C. albicans* constituted the majority of clinical isolates (each genotype: 14.8%). By combining the two typing methods, 27 strains of *C. albicans* were finally divided into 22 genotypes, based on which a phylogenetic dendrogram was constructed by means of DendroUPGMA online software [29] (Figure 3). According to the host and anatomical source of isolation, we could not find any special genotype. However, the limited number of clinical samples in this study restrained the implementation of a proper evaluation. 

**Table 2:** *Candida albicans* susceptibility and minimum inhibitory concentration

**Antifungal drugs**	**MIC range (µg/ml)**	**MIC50 (µg/ml)**	**MIC90 (µg/ml)**	**Resistance (n)**
Fluconazole	0.5-32	2	4	2
Ketoconazole	0.06-2	0.125	0.5	3
Clotrimazole	0.06-2	0.125	0.5	2
Amphotericin B	0.03-0.25	0.125	0.25	0
Nystatin	0.03-0.25	0.125	0.25	0
Caspofungin	0.06-0.5	0.125	0.5	0

n: number

Table 2 summarizes the in vitro susceptibilities of 27 *C. albicans* isolates to fluconazole, clotrimazole, ketoconazole, amphotericin B, and nystatin. Based on the broth microdilution (CLSI M27-S4 document) of 27 tested *C. albicans* isolates, 2 (7.4%), 3 (11%), and 2 (7.4%) strains were resistant to fluconazole (MIC≥8 μg/ml), ketoconazole, and clotrimazole, respectively. The MIC values obtained via broth microdilution method in isolates are presented in Table 2. 

The MIC50 and MIC90 values for fluconazole against *C. albicans* isolates were reported as 8 and 64 µg/ml, respectively. Furthermore, MIC50 and MIC90 values for clotrimazole and ketoconazole were obtained as 0.5 and 2 µg/ml, respectively. The resistance of *C. albicans* isolates to polyene antifungals, including amphotericin B and nystatin, was scarce. Overall, no isolates were resistant to amphotericin B. However, all *Candida* isolates were sensitive to nystatin and caspofungin.

## Discussion

The importance of candidiasis in animals has not been completely recognized yet. However, animal cases of candidiasis have been reported, and the pathogenesis of candidiasis is not as significant as that in humans [16, 30]. Molecular typing methods are significantly useful in the analysis and investigation of the population structure and epidemiology of fungal pathogens, facilitating the perception of infection process in both human and animal populations [18, 31]. 

The current study is one of the first attempts targeted toward the investigation of *C. albicans* isolates collected from domestic animals living in Tehran and Tabriz, Iran, using molecular typing. This research involved the examination of 27* C. albicans* strains isolated from the oral mucosa, skin, and vagina of cats, horses, dogs with cutaneous lesions, cattle with mastitis, and birds with thrush. 

Amplification of 25S rDNA region or ABC typing was accomplished using specific CA-InT primers, measuring the transformative and movable part of intron region (i.e., 25 rDNA). This method is an exclusive and reproducible option for the molecular typing of *C. albicans* [32]. The results led to the identification of genotypes A, B, C, and E. One of the essential features in the determination of the type of 25S rDNA is the ability to distinguish *C. albicans* from *C. dublinienis*, which was not identified in the study (2). Based on the PCR amplification of 25S rRNA, genotype A (40.7%) was the most common genotype among the clinical isolates, followed by genotypes E (22.2%), C (20%), and B (16.6%). 

Our results are similar to those obtained in other studies performed in different countries. Accordingly, genotype A has been reported as the most common genotype in the majority of the studies. *Candida* *albicans* genotype A is the most common group of this *Candida* species in different areas of the world (2). However, *C. albicans* prevalence is different in various studies.

In a study conducted by Ashrafi *et al.* (2014) on the strains isolated from HIV-infected patients, genotype A was identified in 66% of the samples [33]. In a couple of studies performed by Karahan (2004) and Gurbaz (2010) in Turkey on 190 *Candida* strains, nearly 51% of the strains were genotype A [34, 35]. Likewise, in another study carried out in Brazil, Matta *et al.* reported genotype A as the most common genotype [36].

There are limited studies addressing animal strains in this regard. Wrobel *et al.* detected genotype A in 60% of the clinical isolates obtained from animal strains [31]. A significant feature of the present study is the identification of genotype E, which is a new group in *C. albicans* classification using PCR amplification of 25S rDNA. Nonetheless, this genotype has been rarely found in other studies investigating *C. albicans*. Moreover, this kind of genotype was not observed in a study conducted by Ashrafi *et al.* in Iran. It should be noted that the mentioned study was performed on human strains [33]. 

It seems that *C. albicans* population isolated from animals has genetic variation according to the typing of 25S rDNA. However, the achievement of more accurate results requires the investigation of more *Candida* samples from animals. It is expected that group A would be more common in animal isolates similar to human isolates. It is believed that only one molecular typing method fails to draw a complete image of the necessary information required by the investigators. In this regard, ABC typing method, even with the mentioned advantages, cannot provide precise information related to genotypes since it divides the strains into limited groups; as a result, this method was applied, along with other typing systems, in most of the cases. 

In the present study, genotyping based on RPSs was used along with ABC typing. Accordingly, the amplification of ALT regions was performed using specific primers. It has been reported that *C. albicans* chromosomes contain characteristic RPSs, each of which contains a short tandem repeating unit of 172 bp designated as ALT [34, 37]. The number of ALT leads to variations in the molecular sizes of RPSs [38]. The molecular characteristics of different sizes based on the copy numbers of ALT sequences divide *C. albicans* strains into four groups (i.e., Aa, Ab, Ac, and Ad) according to their nucleotide sequences. 

Some specific primers used in this method investigate repetitive sequences and determine the number of the repetitions of ALT regions, such as the well-known P-II primer. Some other ones determine the inner units of ALT regions or those attached to the specific types of ALT like AS-I. In the present study, AS-I primer was adopted. The repetitive element sequence-based PCR with AS-I primer resulted in the identification of 17 specific patterns or genotypes out of 27 strains. These patterns were determined based on the band size formed by the electrophoresis of PCR products. 

In this study, AS1 and AS2 genotypes were more common than other genotypes and had the same frequency. Contrary to such methods as RAPD-PCR, electrophoretic band patterns in the applied method were constant and were less affected by the test conditions and laboratory. AS-1 reflects the arrangement order of ALTs in each RPS unit, and AS-1 primers were used in cases in which the strains were similar in RPS units, but showed diversity with AS-I primers. 

In this study, in order to obtain a detailed and complete pattern for each isolate, the results of ABC typing were compared and aligned with the results obtained through RPS amplification. In this regard, genotypes determined by ABC typing were aligned with those of RPS amplification and led to the identification of 22 genotypes out of 27 strains. This indicated that the applied method had a significant discriminatory power to investigate intraspecific variations that are important from several aspects. 

Identification of 22 different genotypes is notable because it is a rare phenomenon in animals based on previous studies. On the other hand, with regard to the results obtained from molecular typing of the *Candida* species isolated from humans, these findings are not unpredictable. Furthermore, the samples in this study were collected from different animal hosts that could be the reason for this diversity. Certainly, phylogenetic analysis fails to show a clear correlation between clinical cases. There was no specific genotype based on the host; additionally, no relationship was observed between the host and genotype of *C. albicans*. 

It is required to perform more studies on a large number of clinical samples isolated from different animals to obtain clearer results about animal genotypes. The results of the present study showed a genetic diversity in the *Candida* population isolated from animals, indicating a heterogeneous population of *Candida* in animals. These results are similar to those obtained by studies investigating humans, such as those conducted by Wrobel et al. (2008) using multilocus sequence typing method to investigate the genotypes. They evaluated the relationship between human and animal *C. albicans* strains and found that the human-to-animal transmission had a higher probability than animal-to-human transmission [31].

Edelmann et al. (2005), investigating the genetic correlations of *C. albicans* in human and animal strains, achieved a heterogeneous pattern in the strains of *C. albicans* using DNA fingerprinting by Ca_3_ probe. They observed a range of dissociations in the strains of humans and animals [16]. Furthermore, the results of the present study demonstrated the efficiency of molecular typing through investigating RPS regions in *C. albicans*, which can examine intraspecific variation with an appropriate resolution power.

In the current study, we presented data on species distribution and antifungal susceptibility profiles of *Candida* isolates obtained from animals in Iran. In this research, the majority of *Candida* isolates were susceptible to antifungals; however, a low rate of resistance was observed in *C. albicans*. The results revealed no relationship between resistance and any molecular genotypes in the study. 

## Conclusion

It seems that this genotyping system has a high efficiency and can show better results by some modifications. It is suggested to adopt this typing method in order to genotype more animal samples. Future studies are recommended to compare human and animal genotyping by this method. It is also suggested to adopt one particular animal host in different specific geographical areas to explore the correlation between genotypes and pathogenesis. 
